# Green Tea Catechin Epigallocatechin Gallate Inhibits Vegetative Cell Outgrowth and Expression of Beta-Lactamase Genes in Penicillin-Resistant *Bacillus anthracis* Strain PCr

**DOI:** 10.3390/pathogens13080699

**Published:** 2024-08-19

**Authors:** Akiko Okutani, Shigeru Morikawa, Ken Maeda

**Affiliations:** Department of Veterinary Science, National Institute of Infectious Diseases, 1-23-1 Toyama, Shinjuku-ku, Tokyo 162-8640, Japan; morikawa@niid.go.jp (S.M.); kmaeda@niid.go.jp (K.M.)

**Keywords:** *Bacillus anthracis*, green tea catechin, penicillin, beta-lactamase

## Abstract

The green tea catechin epigallocatechin gallate (EGCg) has antimicrobial effects on many bacteria. In this study, we investigated the inhibitory effects of EGCg on *Bacillus anthracis* spores and vegetative cells. The *B. anthracis* spores were insensitive to EGCg, but the growth of vegetative cells derived from germinated spores was inhibited by EGCg. Moreover, EGCg decreased the minimum inhibitory concentration of penicillin and meropenem for penicillin-resistant *B. anthracis*. In the penicillin-resistant *B. anthracis* strain, the transcription levels of the beta-lactamase genes (*bla1* and *bla2*) decreased significantly following the treatment with 50 µg/mL EGCg. These results suggest that the appropriate application of EGCg may effectively control the penicillin-resistant *B. anthracis* growth and beta-lactamase production.

## 1. Introduction

Anthrax, which is caused by the spore-forming bacterium *Bacillus anthracis*, is one of the most dangerous zoonoses, posing a serious threat to both public and animal health. The intentional release of *B. anthracis* spores in the US in 2001 [1] highlighted the growing importance of decontaminating *B. anthracis* spore-contaminated sites [2]. *B. anthracis* spores are resistant to dry and oligotrophic conditions and can survive in the soil for decades, making it extremely difficult to eradicate them from the environment [3]. These spores can cause inhalational, gastrointestinal, or cutaneous anthrax in humans and animals by entering through the lungs, mouth, or skin, respectively. The spores germinate to produce vegetative cells that grow within the host. The vegetative cells are coated with a capsule consisting of γ-linked poly-D-glutamic acid that protects them from the immune cells in the blood [4,5].

*B. anthracis* is susceptible to beta-lactam antibiotics, and penicillin is the antibiotic recommended for postexposure prophylaxis and for the treatment of anthrax cases [6]. Chen et al. [7] reported that 2% to 16% of the isolates were resistant to beta-lactams, and it is known that there are several mutation types within the penicillin-resistant *B. anthracis* genome. The genomic analysis of an anthrax outbreak in cattle revealed microevolution, including penicillin resistance emergence and significant genetic heterogeneity with the presence of multiple SNVs within infected animals [8].

The leaf extract of tea, *Camellia sinensis*, contains polyphenolic compounds with inhibitory effects on a wide spectrum of microbes [9]. Among these components, (−)-epigallocatechin gallate (EGCg) is the most abundant, representing approximately 50% of the catechin pool [10]. An exposure to sub-inhibitory EGCg concentrations for Gram-positive bacteria can reverse the tetracycline resistance in staphylococci by inhibiting the Tet(K) efflux pump; it can also sensitize resistant staphylococci isolates to tetracycline [11] and inhibit the penicillinase activity in penicillinase-producing staphylococci [12]. Among the *Bacillaceae* species, *Bacillus subtilis* is highly sensitive to EGCg, which suppresses the production of bacterial surface proteins [13]. EGCg also affects the foodborne bacterium *Bacillus cereus* [14] and inhibits the activity of the anthrax toxin lethal factor metalloprotease [15]. Green tea and EGCg have bactericidal effects on the encapsulated and non-encapsulated cells of *B. anthracis strain* Ames [16]. Additionally, it can bind to the peptidoglycan of methicillin-resistant *Staphylococcus aureus* (MRSA) and has bactericidal effects [17]. Interactions between EGCg and cell membrane proteins are crucial for the antimicrobial activity of EGCg. Moreover, the synergistic effects of EGCg and beta-lactam antibiotics on MRSA decrease the minimum inhibitory concentration (MIC) of beta-lactams [17]. Although the incidence of beta-lactam antibiotic resistance in *B. anthracis* is not high, the microevolution of penicillin-resistant bacteria was previously confirmed in different parts of the same cattle carcass during an anthrax outbreak [18]. In an earlier study, we analyzed the full genome sequence of the penicillin-resistant *B. anthracis* strain PCr, which was isolated from a cow with anthrax in Japan [19]. In this study, we investigated the inhibitory effects of EGCg on *B. anthracis* spores and vegetative cells, focusing on its potential to address both wild-type and penicillin-resistant strains.

## 2. Materials and Methods

### 2.1. Bacterial Strains and Media

Wild-type *B. anthracis* strain BA103, which is stored at the National Institute of Infectious Diseases (NIID), Tokyo, Japan, was used in this study [18]. Penicillin-resistant *B. anthracis* strain PCr, which was originally isolated in 1978 from a cow in Japan, was kindly supplied by Dr. Makoto Osaki from the National Institute of Animal Health, Ibaraki, Japan [19]. Bacterial cells cultured in Mueller–Hinton (MH) or Luria-Bertani (LB) broth were transferred to the same media solidified with agar for an overnight incubation at 37 °C. EGCg was purchased from Tokyo Kasei Chemical, Tokyo, Japan, whereas benzyl penicillin sodium salt, vancomycin, meropenem, ciprofloxacin, and doxycycline were purchased from Wako Chemical, Osaka, Japan. Live *B. anthracis* cells were cultured at biosafety level 3 according to the Regulations on the Safety Control of Laboratories Handling Pathogenic Agents as authorized by the biological risk management committee of NIID.

### 2.2. Effect of EGCg on B. anthracis Spores

Spores for a germination assay were prepared using a previously reported method that was modified slightly [20]. Briefly, bacteria were grown on Schaeffer’s sporulation agar medium at 30 °C for 5 days, after which spores were collected using ice-cold distilled water. Residual vegetative cells were killed by heating at 65 °C for 20 min. To remove vegetative cell debris, the spores were washed multiple times with ice-cold distilled water. A phase contrast microscope was used to assess spore purity (>99%). To determine the sensitivity of *B. anthracis* spores to EGCg, a suspension containing 1 × 10^6^ spores was mixed with EGCg at a final concentration of 0 or 100 µg/mL and then added to MH broth prior to a 24 h incubation at 37 °C. The optical density at 600 nm (OD_600_) was measured at 0, 10, 20, 30, 40, 50, 60, 70, 80, 90, and 120 min as well as at 2, 4, 6, 8, and 24 h following inoculation. The decrease in OD_600_ (relative to the initial value at 0 min) was calculated for each time point.

### 2.3. Time-Kill Kinetics Assay and MIC Determination

A time-kill kinetics assay of EGCg treatment effects was performed and the MIC for the combined treatment with antibiotics and EGCg was determined according to a broth dilution method [21]. Bacterial cells (>1 × 10^5^ colony-forming units (CFU)/mL) from an overnight MH broth culture were transferred to 3 mL MH broth containing EGCg (0, 25, 50, or 100 µg/mL) and incubated at 37 °C for 27 h. During this incubation period, 5 µL medium was collected at 0, 3, 6, 9, 12, and 27 h, diluted (1:10), and added to LB agar medium, which was then incubated at 37 °C for 24 h. The colonies on the LB agar medium were counted to calculate CFU/mL.

### 2.4. Beta-Lactamase Production and Quantitative Real-Time Polymerase Chain Reaction (qRT-PCR) Analysis

Beta-lactamase production was assessed using BD BBL Cefinase β-Lactamase Detection Discs (Thermo Fisher Scientific, Waltham, MA, USA) according to the manufacturer’s instructions. Briefly, 10 μL droplets of 24 h cultures of penicillin-resistant *B. anthracis* strain PCr and penicillin-susceptible wild-type strain BA103 in MH broth supplemented with EGCg (0, 10, 50, 100, or 500 μg/mL) were applied to Cefinase discs. A color change (yellow to red) after 10 min indicated beta-lactamase was produced.

Strains BA103 and PCr were also cultured in 3 mL MH broth with or without EGCg (12.5, 25, or 50 µg/mL) for 1.5, 3, and 6 h (exponential growth phase) or 24 h (stationary phase). Total RNA was extracted using TRIzol (Thermo Fisher Scientific, Waltham, MA, USA) according to the manufacturer’s instructions. Purified total RNA (20 ng) was used for a qRT-PCR analysis, which was performed using a One-Step SYBR PrimeScript II RT-PCR kit (TaKaRa Bio Inc., Otsu, Shiga, Japan) and a LightCycler 480 real-time PCR system (Roche Molecular Diagnostics, Santa Clara, CA, USA). Gene-specific primers were designed for *bla1* (forward: 5′-CCGGGTTACTATGTCTGATCGTT-3′ and reverse: 5′-TATGATTTGGAAGCGCATTTCC-3′), *bla2* (5′-GGCAGAAAAGAAATTTAAGAAGAGTGTAA-3′ and 5′-TTAATGCCTCTTTCTTTCAACGTTT-3′), and the internal control gene GAPDH (*gapA*) (5′-AACAGCGCCTGGTAAAAATG-3′ and 5′-TCACAACAGGCGCTAAACAG-3′). To synthesize cDNA, RNA was reverse transcribed at 42 °C for 5 min, after which the polymerase was activated at 95 °C for 10 s. A three-step PCR was completed as follows: 40 cycles of 95 °C for 5 s, 60 °C for 20 s, and 72 °C for 40 s.

### 2.5. Statistical Analyses

Experiments were performed in triplicate. Data are expressed herein as the mean ± standard deviation. A one-way analysis of variance (ANOVA) with post hoc Tukey’s multiple-comparison tests or a two-way ANOVA followed by Tukey’s, Dunnett’s, or Sidak’s multiple-comparison tests were performed to analyze data. Statistical analyses were conducted using GraphPad Prism 9, version 9.3.1 (GraphPad Software, San Diego, CA, USA).

## 3. Results

### 3.1. EGCg Decreased the MIC of Antibiotic Treatments of Penicillin-Resistant B. anthracis Strain PCr

**Figure 1:**
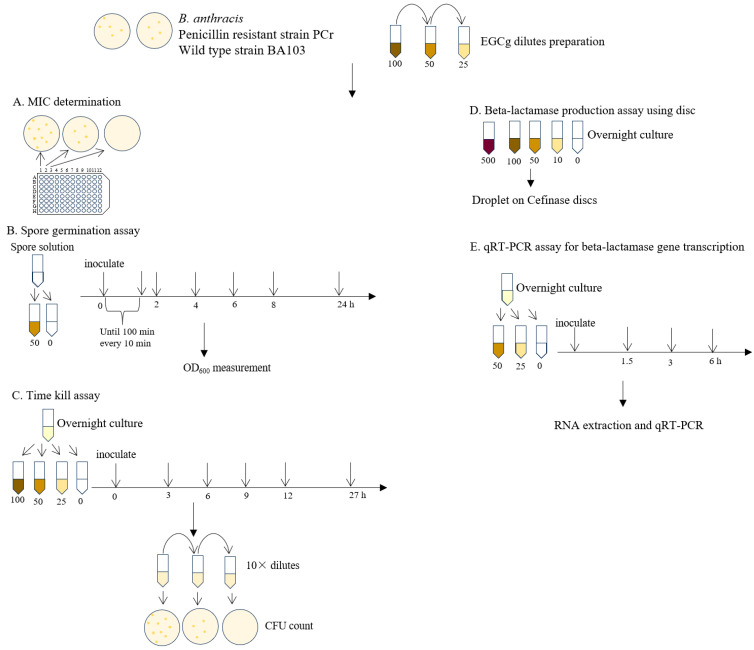
Schematic overview of experimental design for investigating EGCg effects on *B. anthracis* of MIC determination, spore germination studies, time-kill assays, and beta-lactamase activity and gene expression analysis.

Schematic overview of the experiments in this study is shown in Figure 1.

The MICs of the antibiotics combined with EGCg for the penicillin-resistant *B. anthracis* strain PCr were determined using a broth dilution method (Table 1). The MIC of penicillin (beta-lactam) for the strain PCr was the highest (>800 µg/mL) in the absence of EGCg, but it decreased in a dose-dependent manner when EGCg was included in the treatment, reaching ˂100 µg/mL with 100 µg/mL EGCg (Table 1). The MIC of meropenem (carbapenem) for the strain PCr was the highest (>1024 µg/mL) when EGCg was not included, but it decreased to ˂64 µg/mL in the presence of 100 µg/mL EGCg. The addition of EGCg also affected the MICs of penicillin and meropenem for the wild-type *B. anthracis* strain BA103 when comparing the MIC values at 25 vs. 50 µg/mL EGCg (Table 1). While the absolute MIC values for BA103 were lower than those for the PCr strain, the relative reduction in MIC (approximately 10-fold) was similar for both strains.

### 3.2. Inhibitory Effect of EGCg on B. anthracis Vegetative Cell Outgrowth

**Figure 2:**
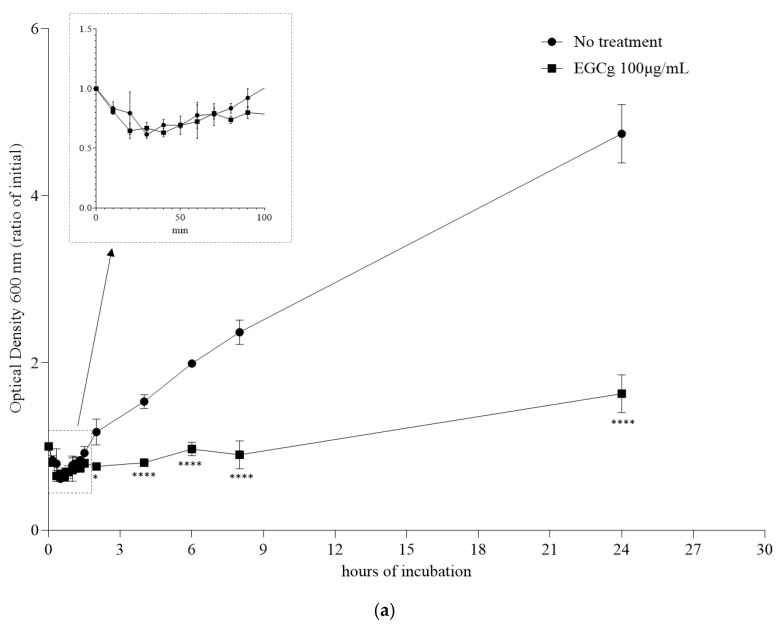

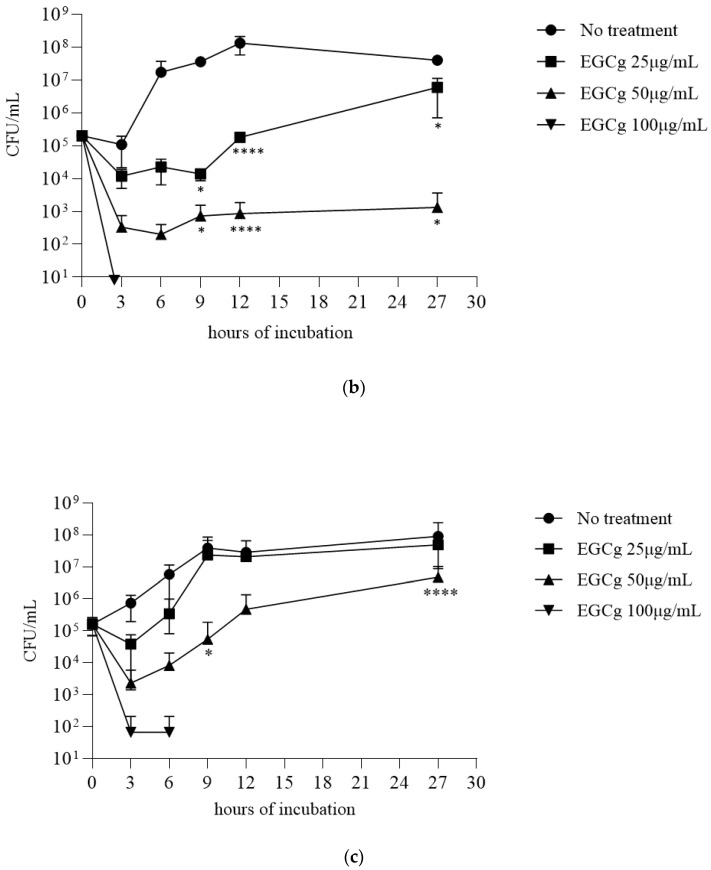
(**a**) Growth curve from spores of penicillin-resistant *Bacillus anthracis* strain PCr treated without EGCg (no-treatment control) or with 100 μg/mL EGCg. The optical density at 600 nm (OD_600_) was calculated (relative to the initial OD_600_). Significant differences from the no-treatment control are indicated by asterisks: * *p* < 0.05 and **** *p* < 0.0001. The graph inset presents the decrease in OD_600_ due to spore germination. (**b**) Growth curve of vegetative cells of penicillin-resistant *B. anthracis* strain PCr treated with EGCg (25, 50, and 100 μg/mL). Colony-forming unit (CFU)/mL was calculated after 3, 6, 9, 12, and 27 h incubations. Significant differences from the no-treatment control are indicated by asterisks: * *p* < 0.05 and **** *p* < 0.0001. (**c**) Growth curve of vegetative cells of wild-type *B. anthracis* strain BA103 treated with EGCg (25, 50, and 100 μg/mL). Colony-forming unit (CFU)/mL was calculated after 3, 6, 9, 12, and 27 h incubations. Significant differences from the no-treatment control are indicated by asterisks: * *p* < 0.05 and **** *p* < 0.0001.

We examined the inhibitory effect of EGCg on the *B. anthracis* strain PCr spore germination and vegetative cell outgrowth at 37 °C in MH broth supplemented with 100 µg/mL EGCg. The spore germination was determined according to the decrease in the OD_600_ value within 100 min following the inoculation [21]. The decrease in OD_600_ was the same for the EGCg treatment and the no-treatment control, suggesting that EGCg did not inhibit the spore germination (Figure 2a). However, after the spores germinated, the vegetative cell outgrowth was inhibited by EGCg after a 24 h incubation (Figure 2a). We then completed a time-kill assay to clarify how EGCg affects the strain PCr and BA103 vegetative cells derived from the germinated spores. Specifically, the MH broth was inoculated with spores for an overnight culture, after which EGCg was added prior to a 27 h incubation. The 25, 50, and 100 µg/mL EGCg treatments significantly inhibited the vegetative cell growth (Figure 2b,c). More specifically, the bacterial growth was undetectable in the MH broth containing 100 µg/mL EGCg after a 3 h incubation of PCr and a 6 h incubation of BA103. The vegetative cell growth was significantly inhibited in the MH broth containing 50 µg/mL EGCg after a 27 h incubation for both strains. The 25 µg/mL EGCg treatment resulted in slightly delayed vegetative cell outgrowth for the first 9 h of the incubation period. After a 27 h incubation, the cell growth rates were still lower for the 25 µg/mL EGCg treatment than for the no-treatment control.

### 3.3. Beta-Lactamase Production in Penicillin-Resistant B. anthracis Strain PCr

**Figure 3:**
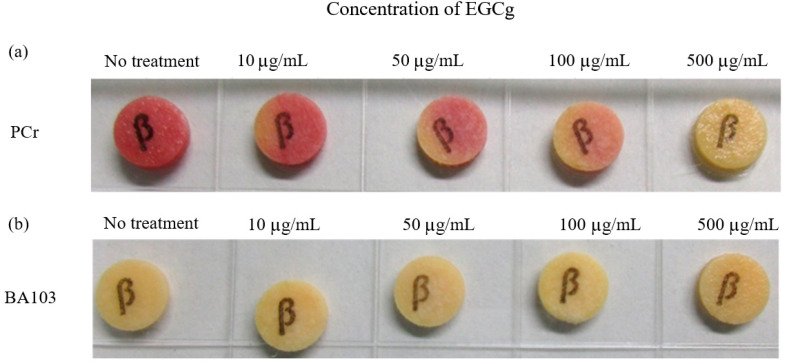
Production of β-lactamase in penicillin-resistant and wild-type *Bacillus anthracis*. Overnight cultures of penicillin-resistant *B. anthracis* (PCr) (**a**) and wild-type *B. anthracis* (BA103) (**b**) in MH broth supplemented with EGCg (0, 10, 50, 100, and 500 μg/mL) were added to Cefinase discs. The production of β-lactamase was indicated by a change in disc color (yellow to red). The intensity of the color change reflected the extent of β-lactamase production.

**Figure 4:**
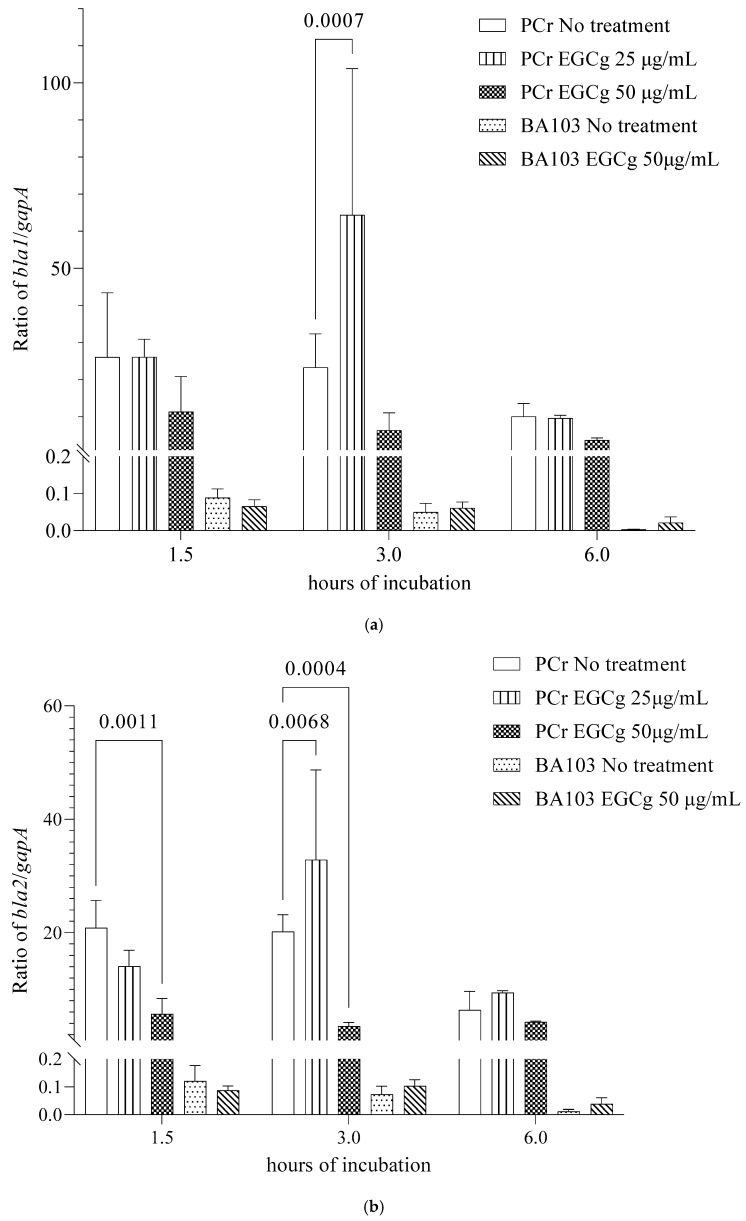
Analysis of β-lactamase gene (*bla1* and *bla2*) expression in *Bacillus anthracis* PCr grown in MH broth supplemented with EGCg (25 and 50 µg/mL) and in *B. anthracis* BA103 with EGCg 50 µg/mL. The *bla1* (**a**) and *bla2* (**b**) mRNA levels after 1.5, 3, and 6 h incubations were determined by qRT-PCR and normalized against *gapA* mRNA levels. The mean and standard deviation were calculated from two independent experiments. Significant differences between EGCg treatments and the no-treatment control are indicated.

We analyzed the beta-lactamase production in the *B. anthracis* strain PCr using Cefinase discs (Figure 3a). The addition of EGCg in the MH broth inhibited the beta-lactamase production in the strain PCr in a dose-dependent manner, whereas the beta-lactamase production was undetectable in wild-type strain BA103 (Figure 3b). Because the inhibitory effects of EGCg were detectable immediately after the strain PCr vegetative cells were treated with EGCg (Figure 2b), we investigated whether EGCg affected the transcription levels of the *B. anthracis* beta-lactamase genes *bla1* and *bla2* in the very early part of the exponential growth phase (1.5 h after starting the culture), early part of the exponential growth phase (3 h), and middle part of the exponential growth phase (6 h). Compared with the corresponding levels in the no-treatment control, the *bla1* and *bla2* transcription levels increased significantly in the strain PCr treated with 25 µg/mL EGCg at the 3 h time point, and the *bla2* transcription levels at the 1.5 and 3 h time points differed significantly when treated with 50 µg/mL EGCg (Figure 4). In the wild-type strain BA103, both genes were transcribed at extremely low levels.

## 4. Discussion

Our study investigated the antimicrobial effects of EGCg on penicillin-resistant *B. anthracis*, representing a significant advancement in our understanding of EGCg’s antimicrobial properties. While the previous studies have explored EGCg’s effects on various pathogens, our work is the first to comprehensively investigate its impact on both wild-type and penicillin-resistant *B. anthracis* strains, including its effects on spore germination, vegetative growth, and beta-lactamase gene expression.

We found that EGCg inhibits vegetative cell outgrowth without affecting spore germination, decreases the minimum inhibitory concentration (MIC) of beta-lactam antibiotics, and suppresses the expression of beta-lactamase genes in both wild-type and penicillin-resistant *B. anthracis* strains. These findings align with and extend the previous research on EGCg’s antimicrobial properties. Reygaert et al. [22] demonstrated the efficacy of green tea catechins, including EGCg, against various pathogens, and our study extends this to penicillin-resistant *B. anthracis*. The synergistic effect between EGCg and beta-lactam antibiotics observed in our study is consistent with the previous findings in methicillin-resistant *Staphylococcus aureus* (MRSA) [11,12,17].

Xu et al. [23] showed that EGCg suppresses the cell-wall-related gene expression in *Streptococcus mutans*, which aligns with our observation of beta-lactamase gene suppression in *B. anthracis*. Our finding that EGCg does not inhibit spore germination but affects vegetative cell outgrowth is a novel contribution to the field, with significant implications for antimicrobial strategies. This selective action may contribute to the synergistic effects observed with beta-lactam antibiotics, potentially increasing the window of vulnerability for *B. anthracis* cells transitioning from spores to the vegetative state.

Interestingly, our analysis revealed a dose-dependent effect of EGCg on beta-lactamase gene expression and production. At higher concentrations (50 µg/mL), EGCg decreased the beta-lactamase production, which correlated with the suppressed transcription of the beta-lactamase genes (Figure 4). However, at lower concentrations (25 µg/mL), we observed an increase in the beta-lactamase gene transcription during the early exponential growth phase despite no detectable increase in the beta-lactamase production (Figure 3). The vegetative cells of the penicillin-resistant strain PCr were found to be significantly more sensitive to EGCg at 50 µg/mL compared to the wild-type BA103 (Figure 2b,c). This approximately 1000-fold difference in sensitivity suggests that EGCg may have a more pronounced effect on antibiotic-resistant strains. This finding has important implications for the potential therapeutic applications of EGCg, particularly in combating antibiotic-resistant *B. anthracis* infections. It also underscores the need for further investigation into the mechanisms underlying this differential sensitivity. This complex relationship suggests that EGCg’s effects on *B. anthracis* may involve multiple mechanisms and highlights the importance of careful dosage consideration in potential therapeutic applications.

Our investigations also included higher concentrations of EGCg (200, 400, and 800 µg/mL), which resulted in the complete inhibition of the bacterial growth for all the strains tested. While these concentrations may not be practical for therapeutic applications due to potential toxicity concerns, they demonstrate the potent antimicrobial activity of EGCg at higher doses and establish an upper limit for its effectiveness against *B. anthracis* in our experimental setup. These findings align with broader studies on EGCg’s antimicrobial properties [24,25].

The inhibition of the beta-lactamase gene expression by EGCg may explain the synergistic effects observed with beta-lactam antibiotics for both the wild-type and penicillin-resistant strains. This mechanism suggests that EGCg could potentially restore the effectiveness of penicillin and related antibiotics against resistant strains. Notably, the effect of EGCg on the MIC reduction was similar (approximately 10-fold) for both the wild-type and penicillin-resistant strains, although the absolute MIC values for the wild-type strain were lower. This synergistic effect has been observed in other bacterial species as well [26].

Our experiments with prolonged incubation times and serial passages did not reveal the emergence of EGCg-resistant mutants. The transient growth observed at certain time points (9 and 27 h) in the presence of EGCg may be attributed to temporary adaptations or heterogeneity in the bacterial population’s susceptibility rather than true resistance development. This finding is significant as it suggests that EGCg may have a low potential for inducing resistance, a crucial factor for its long-term efficacy [22] as an antimicrobial agent against *B. anthracis*.

The selective action of EGCg on vegetative cells rather than spores could be advantageous in both clinical and environmental applications. In clinical settings, EGCg could serve as an adjuvant therapy, potentially lowering the effective dose of antibiotics and reducing side effects. Environmentally, EGCg’s ability to inhibit vegetative cell outgrowth without affecting spore germination could be valuable in controlling the *B. anthracis* proliferation in contaminated areas.

The known effects of EGCg on bacterial toxins, combined with our findings, suggest that EGCg could target both bacterial growth and toxin-mediated pathogenesis. Dell’Aica et al. [15] reported that EGCg inhibits the anthrax lethal factor, a key component of the anthrax toxin. Additionally, Shimamura et al. [27] revealed that catechins, particularly EGCg, can directly bind to *Staphylococcal* enterotoxin A (SEA), primarily through electrostatic and hydrophobic interactions. These findings suggest that EGCg may have broader applications in neutralizing various bacterial toxins beyond just inhibiting their production [24].

Several limitations of our study should be acknowledged. Our experiments were conducted in vitro, and the efficacy of EGCg in vivo remains to be determined. The complex environment within a host organism may affect EGCg’s bioavailability and activity. While we demonstrated EGCg’s ability to inhibit beta-lactamase gene expression, the precise molecular mechanism underlying this inhibition requires further investigation. The long-term effects of EGCg treatment, including the potential for resistance development over extended periods, were not fully assessed in this study.

In conclusion, our study provides compelling evidence for the potential utility of EGCg as part of a novel approach to managing wild-type and antibiotic-resistant *B. anthracis* strains. The demonstrated ability of EGCg to inhibit vegetative cell outgrowth, enhance antibiotic efficacy, and suppress beta-lactamase gene expression opens new avenues for both medical treatments and biodefense strategies against *B. anthracis* [28].

While further research is needed, particularly in vivo studies and investigations under various environmental conditions, these findings represent a significant step forward in our understanding of EGCg’s antimicrobial properties. The future studies should focus on optimizing the EGCg formulations for clinical and environmental applications, assessing the long-term efficacy and resistance development, and further elucidating the molecular mechanisms of EGCg’s action against *B. anthracis*.

## Figures and Tables

**Table 1:** MICs of antibiotics combined with EGCg in treatments of penicillin-resistant *B. anthracis* PCr and wild-type BA103.

EGCg (µg/mL)	MIC of Penicillin (µg/mL)		MIC of Meropenem (µg/mL)	
	PCr	BA103	PCr	BA103
0	>800	0.125	>1024	0.063
25	>800	0.25	256	0.063
50	100	0.016	128	0.031
100	<100	*	<64	*

*: no growth was observed in the media.

## Data Availability

The data used to support the findings of this study are included in the article.
